# Variables affecting penetrance of gastric and duodenal phenotype in familial adenomatous polyposis patients

**DOI:** 10.1186/s12876-018-0841-8

**Published:** 2018-07-16

**Authors:** Danielle C. Sample, N. Jewel Samadder, Lisa M. Pappas, Kenneth M. Boucher, Wade S. Samowitz, Therese Berry, Michelle Westover, Deepika Nathan, Priyanka Kanth, Kathryn R. Byrne, Randall W. Burt, Deborah W. Neklason

**Affiliations:** 1 Huntsman Cancer Institute, University of Utah, 2000 Circle of Hope, Salt Lake City, UT 84112-5550 USA; 2 Division of Gastroenterology, University of Utah, Salt Lake City, UT USA; 3 Division of Epidemiology in the Department of Internal Medicine, University of Utah, Salt Lake City, UT USA; 4 Department of Pathology, Huntsman Cancer Institute, University of Utah, 2000 Circle of Hope, Room 3100, Salt Lake City, UT 84103 USA; 5 University of Utah School of Medicine, 175 North Medical Drive East, Salt Lake City, UT 84132 USA; 6 Department of Clinical Genomics and Gastroenterology, 5777 E Mayo Blvd, Phoenix, AZ 85054 USA; 7 University of California Irvine, 333 City Blvd W, St 800, Irvine, CA 92868 USA; 8 University of Utah School of Medicine, 30 N 1900 E, Salt Lake City, UT 84132 USA; 9 Hereditary Gastrointestinal Cancer Registry, Huntsman Cancer Institute at University of Utah, 2000 Circle of Hope, Salt Lake City, UT 84112-5550 USA

**Keywords:** Duodenum, Gastric, Polyposis, Familial adenomatous polyposis, Fundic gland polyps

## Abstract

**Background:**

Patients with familial adenomatous polyposis (FAP) frequently undergo colectomy to reduce the 70 to 90% lifetime risk of colorectal cancer. After risk-reducing colectomy, duodenal cancer and complications from duodenal surgeries are the main cause of morbidity. Our objective was to prospectively describe the duodenal and gastric polyp phenotype in a cohort of 150 FAP patients undergoing pre-screening for a chemoprevention trial and analyze variables that may affect recommendations for surveillance.

**Methods:**

Individuals with a diagnosis of FAP underwent prospective esophagogastroduodenoscopy using a uniform system of mapping of size and number of duodenal polyps for a 10 cm segment. Gastric polyps were recorded as the total number.

**Results:**

The distribution of the count and sum diameter of duodenal polyps were statistically different in two genotype groups, those with *APC* mutations associated with classic FAP had a greater count (median 17) and sum diameter of polyps (median 32 mm) than those with *APC* mutations associated with attenuated FAP (median count 4 and median sum diameter of 7 mm) (*p* < 0.0001). The number of gastric polyps did not differ based on genotype (*p* = 0.67) but advancing age correlated with severity of gastric polyposis (*p* = 0.019). Spigelman (modified) staging of II or greater was found in 88% of classic FAP patients and 48% attenuated FAP patients. Examples of severe and mild upper GI phenotype are observed in patients with identical APC mutations, showing that the APC mutation location is not absolutely predictive of an upper GI phenotype.

**Conclusions:**

Most FAP patients have duodenal and gastric polyps which become more prevalent and advanced with age. Standard upper endoscopic surveillance is recommended based on personal history independent of *APC* mutation location.

**Trial registration:**

NCT 01187901 registered August 24, 2010, prospective to enrollment.

## Background

Familial adenomatous polyposis (*APC* [MIM 175100]) is an autosomal dominant, colon cancer predisposition syndrome, characterized by the presence of hundreds to thousands of adenomatous polyps in the colon and nearly a 100% risk of developing colon cancer if the colon is not removed [1]. Mutations in the *APC* gene are the most common cause of this syndrome with a prevalence estimated at 1:10,000 persons [2]. Mutation carriers may also present with polyps in the upper gastrointestinal (GI) tract and have an increased risk of gastric, small bowel, thyroid, brain, and other malignancies. Extracolonic features can include duodenal adenomas, gastric fundic gland polyps with antral sparing, osteomas, desmoid tumors, dental anomalies, cutaneous lesions and congenital hypertrophy of the retinal pigment epithelia (CHRPE). As increasing numbers of prophylactic colectomies are performed to decrease colorectal cancer risk, desmoids and duodenal cancer are the main cause of morbidity in patients with FAP [3]. Duodenal adenomas are estimated to occur in 50–90% of patients with FAP, with approximately 5% of duodenal polyps progressing to cancer [4, 5]. Lifetime risks of duodenal and gastric cancers are estimated to be 4–12 and < 1% respectively.

The genomic position of the *APC* mutation has been correlated with profuse, intermediate and attenuated colonic polyposis. To add to this spectrum of variable phenotype, recent report found no colonic polyposis with a specific point mutation in the *APC* promoter 1B YY1 transcription factor binding site in hereditary gastric adenocarcinoma and proximal polyposis of the stomach (GAPPS) [6, 7]. Current guidelines recommend initiation of colonic surveillance at age 10 to 12 for those with a genomic *APC* mutation consistent with profuse and intermediate polyposis (also called classic FAP) and in the late teens for attenuated familial adenomatous polyposis (AFAP) [8]. Unlike colon surveillance, current guidelines for upper GI surveillance do not distinguish between *APC* genotype; baseline upper endoscopy with side viewing is recommended starting at age 20 to 25 with a frequency ranging from 3 to 48 months as determined by the extent of duodenal polyposis (NCCN Guidelines Version 2.2015) [9]. Spigelman staging is a common standard for describing the extent of duodenal polyposis and is based on size, number, histologic type and dysplasia [10]. Spigelman staging has been used to describe the prevalence and progression of duodenal adenomas and cancer in classic FAP resulting in current guidelines for clinical management [4]. There are reports of upper GI phenotype in individuals with mutations associated with attenuated familial adenomatous polyposis but the studies have been limited by their retrospective design, small patient cohorts and vary dramatically in results [11–14]. Due to the limited and conflicting evidence in the literature we prospectively documented the upper GI phenotype in 150 FAP patients undergoing screening for a chemoprevention trial (NCT 01187901). Seventy one of these had an *APC* mutation consistent with the attenuated colonic polyposis form and 79 had an *APC* mutation consistent with intermediate or profuse colonic polyposis. We analyzed the upper GI phenotype in relation to age, gender, age when colectomy was performed and location of *APC* mutation.

## Methods

### Study population

All aspects of this study were approved by University of Utah’s Institutional Review Board for human subject research. Research participants included individuals undergoing baseline endoscopy to determine eligibility for a chemoprevention study of duodenal polyposis in FAP [15]. The study was conducted at a single academic center from July 2010 to June 2014, registered with https://clinicaltrials.gov/ as NCT 01187901 on August 24, 2018 with a study start date of April, 2010. First participant was enrolled and underwent research endoscopy September, 2010. All esophagogastroduodenoscopies (EGDs) were scheduled within a time interval consistent with their individual clinical recommendation. Eligibility criteria required participants to be between 18 and 69 years of age, have either a pathologic mutation of the APC gene (genetic diagnosis) or a phenotype consistent with classic FAP (> 100 colonic adenomas, and additional evidence including self-reported genetic test, colectomy at a young age and additional affected family members), an intact duodenum, absence of severe or uncontrolled medical condition (s), minimum of three years from cancer diagnosis and discontinued use of nonsteroidal anti-inflammatory drugs for 28 days.

### Endoscopy procedures and data capture

Data were collected prospectively. All EGD procedures were medically indicated based on patient’s medical history of past EGD procedures, although the timing since the last EGD procedure ranged from 6 months to this being the first procedure. EGDs were completed using a uniform system of mapping for the 10 cm duodenal segment. In order to obtain an accurate examination during endoscopy, multiple passes with pullbacks were used to get the correct orientation, distance, and counts. This included successive pullbacks with insertion back to polyps just identified to ensure accuracy and allow for further examination. A tattoo was placed 10 cm distal to the duodenal bulb to define the region of study (duodenum proximal to the tattoo). A side viewing endoscope was used as needed to examine the Ampulla of Vater. The maximum diameter and location of each duodenal polyp were recorded in 10 segments, each representing 1 cm of the duodenum, between the duodenal bulb and the tattoo. The diameter of polyps was estimated using the 7 mm opening of forceps. During the procedure, the specific number of duodenal polyps and sum diameter of duodenal polyps (diameter of each polyp observed in the 10 cm segment of duodenum added together) were recorded for each patient. A modified Spigelman classification was used to stage the severity of duodenal polyposis. Points were assigned for polyp number and maximum size using standard Spigelman criteria [10]. Polyps were only biopsied for histologic evaluation if they were > 10 mm or had a clinically concerning appearance. Thus, in the absence of histologic evaluation, polyps ≤10 mm in size were assigned points associated with tubular adenoma and mild dysplasia for Spigelman classification as ~ 90% of colonic polyps and ~ 95% of duodenal polyps ≤10 mm are found to be tubular adenomas [16, 17]. In this study, 12 polyps (1 at < 5 mm, 6 at 5-10 mm, 3 at 11-20 mm, and 1 at 50 mm) required biopsy at baseline. All but one 10 mm and one 50 mm polyp were found to be tubular adenomas, further supporting the modified Spigelman classification applied here. Gastric polyps were recorded as the total number and the size range as defined by the endoscopist.

### Statistical analysis

FAP and AFAP groups were compared to determine how the groups differed prior to inclusion in the clinical trial. Differences in gender, genetic diagnosis, colectomy, nonsteroidal anti-inflammatory drug (NSAID) use, and current smoker were determined by Chi-square test. Number of duodenal polyps, sum diameter of duodenal polyps, and number of gastric polyps were continuous variables, summarized with median and quartiles, and differences in distribution between FAP and AFAP groups were assessed with a Wilcoxon test. Associations of duodenal sum diameter and gastric polyp count with gender and age at endoscopy were estimated for all participants by Kruskal-Wallis tests and Spearman’s rank statistics respectively.

All authors had access to the study data and reviewed and approved the final manuscript.

## Results

### Patient demographics

A total of 385 potential participants were prescreened: 229 were excluded and 156 were enrolled in the study. Of those excluded, 32 had undergone duodenectomy or had advanced histology (27 FAP and 5 AFAP) and 7 had a documented history of a recent absence of duodenal polyps (3 FAP and 4 AFAP). The remaining 190 were excluded due to health problems (92), lack FAP diagnosis (11), outside age range (54), medication intolerance or contraindication (21), EGD performed in the last 6 months (1), or other personal reasons (11). One hundred fifty subjects with complete endoscopy data were included in the analysis with an average age of 41.4 (median 40, range 18–68) years and 42% being male gender. This included 79 FAP patients, at an average age of 39.6 (range 18–67) years and 71 AFAP patients at an average age of 43.4 (range 18–68) years (Table 1). All 71 AFAP subjects and 69 of the 79 FAP patients had a genetic diagnosis or clinical diagnosis with a known family mutation. The remaining 10 FAP patients had a clear clinical diagnosis. A total of 102 unique families (defined as <4th degree relationship between individuals) were represented and included 7 quartets, 7 trios, and 13 pairs of relatives. The age at colectomy was statistically different between the two groups as expected. Because NSAID use within the past 3 months and tobacco smoking could modify the clinical presentation, we considered and adjusted for the frequency of these behaviors (11% NSAID users and 15% smokers; Table 1). This was not significantly different between the FAP and AFAP subsets.

**Table 1:** Demographics of FAP and AFAP study participants

Characteristics	Total (*n* = 150)	FAP (*n* = 79)	AFAP (n = 71)	*p*-value^1^
Male (%)	42% (*n* = 62)	41% (*n* = 32)	42% (*n* = 30)	0.8282
Age in years				
Mean	41.4	39.6	43.4	0.0533
Median	40	38.0	44	
Range	18–68	18–67	18–68	
Genetic diagnosis	93.3%	87.3%	100%	
Colectomy	73.3% (*n* = 110)	98.7% (*n* = 78)	45.0% (*n* = 32)	< 0.0001
Average age at colectomy (range)	28.1 (8–60)	23.4 (8–46)	39.4 (22–60)	< 0.0001
NSAID usage in last 3 months	11.3% (*n* = 17)	13.9% (*n* = 11)	8.5% (*n* = 6)	0.2910
Current smokers	14.7% (*n* = 22)	15.2% (*n* = 12)	14.1% (*n* = 10)	0.8490
Number unique families	68.0% (*n* = 102)	75.9% (*n* = 60)	59.2% (*n* = 42)	

^1^Categorical values compared with chi-square test (gender, genetic diagnosis, colectomy, NSAID use, current smokers). Continuous variables compared with Wilcoxon rank sum test (age, average age at colectomy)

### Upper GI phenotype

**Fig. 1:**
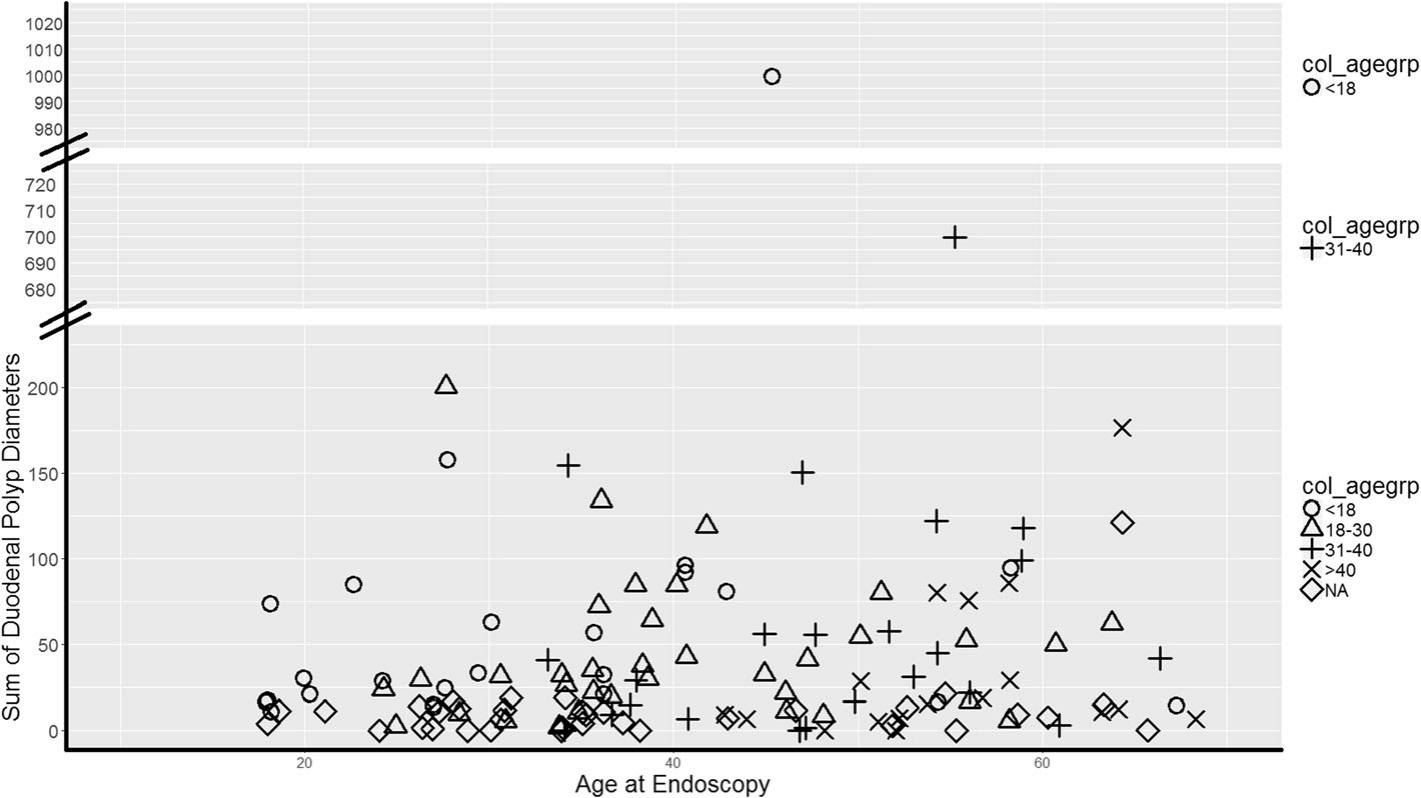
Sum diameter of duodenal polyps grouped by age at colectomy. The study consisted of 110 subjects with a colectomy and divided into 4 age groups and one group that had not had colectomy (25 juvenile at age 8–17 years indicated by (○); 38 young adult at age 18–30 years indicated by (Δ); 29 adult at age 31–40 years indicated by (+) and 18 mature adult at age 41 or older indicated by (x)) and 40 who had not undergone a colectomy at time of upper endoscopy (NA) indicated by (◊). Each individual is plotted based on duodenal polyp burden in millimeters versus the age when the duodenal data were captured by endoscopy

The documented 10 cm segment distal to the duodenal bulb was generally representative of the adenoma burden more distal to the 10 cm position tattoo marker. Most patients had one or more duodenal polyps (92%), with a median of 7 polyps and a sum diameter of 15 mm (Table 2). The number and sum diameter of duodenal polyps were higher in classic FAP versus AFAP patients (*p* < 0.0001; Table 2). The stage of duodenal polyposis, based on a modified Spigelman criteria as described in Methods, was also more severe in the classic FAP patients as compared with the AFAP patients (*p* < 0.0001). The majority of AFAP patients were stage 0, I and II, and the majority of classic FAP patients were stage II and III (Fig. 1). Most of the advanced staging was due to number of polyps. Only two individuals had a duodenal adenoma with advanced histology (tubulovillous): one FAP (50 mm polyp) and one AFAP (10 mm polyp). Two AFAP patients had polyps ≥10 mm (10 and 12 mm), and four FAP patients had polyps ≥10 mm (10, 14, 20, and 50 mm). As detailed in Table 2, polyps were observed on the ampulla in 20 of 117 individuals (15 FAP and 5 AFAP); 6 were biopsied, 4 were tubular adenomas (3 FAP and 1 AFAP) and 2 were normal mucosa (1 FAP and 1 AFAP). Additionally, 3 individuals had undergone an ampullectomy prior to this study (2 FAP and 1 AFAP). In summary, the duodenal phenotype was more severe in FAP versus AFAP as measured by number of polyps, sum diameter of polyps or modified Spigelman stage.

**Table 2:** Comparing FAP versus AFAP upper gastrointestinal phenotypes

Characteristics	Total (*n* = 150)	FAP (*n* = 79)	AFAP (*n* = 71)	*p*-value^1^
Number duodenal polyps:				
Median (25th–75th percentile)	7 (1–18)	17 (8–33)	4 (0–7)	< 0.0001^*^
Sum diameter duodenal polyps:				
Median (25th–75th percentile)	15.5 (5–43)	32 (15–75)	7 (0–15)	< 0.0001^*^
Number with zero duodenal polyps	27 (18.0%)	6 (7.6%)	21 (29.6%)	0.0005
Spigelman classification: 0	27 (18.0%)	6 (7.6%)	21 (29.6%)	< 0.0001^*^
I	19 (12.7%)	3 (3.8%)	16 (22.5%) 30 (42.3%)	
II	81 (54%)	51 (64.6%)		
III	22 (14.7%)	18 (22.8%)	4 (5.6%)	
IV	1 (0.7%)	1 (1.3%)	0 (0%)	
Number of ampullas with adenoma involvement:				
Yes	20 (13.3%)	15 (19%)	5 (7%)	0.0659
No	97 (64.7%)	50 (63.3%)	47 (66.2%)	
Missing	33 (22%)	14 (17.7%)	19 (26.8%)	
Sum diameter duodenal polyps ≥10 mm	65% (*n* = 97)	82% (*n* = 67)	44% (*n* = 30)	< 0.0001^*^
Number gastric polyps:				
Median (25th–75th percentile)	72.5 (15–200)	50 (15–150)	100 (1–200)	0.6703
Patients with > 10 gastric polyps	78.0% (117)	81.0% (64)	74.7% (53)	0.3474

^1^Continuous variables compared with Wilcoxon rank sum test (number and sum diameter of polyps). Median and 25th–75th percentile describe the distribution of the variable within the Total, FAP and AFAP groups. Categorical values compared with chi-square test (sum diameter duodenal polyps > 10, patients > 10 gastric polyps).

^*^When smokers or recent NSAID usage was excluded, there was no change in significant associations with FAP vs AFAP

The majority (78%) of patients also had gastric polyposis, defined as > 10 gastric polyps. There were no significant differences between the FAP and AFAP groups in number of gastric polyps (FAP median = 50, AFAP media*n* = 100, *p* = 0.67) or proportion of patients with > 10 gastric polyps (Table 2). The majority of gastric polyps biopsied were fundic gland. The exceptions were 3 individuals with tubular adenomas (2 FAP, 1 AFAP) and one with a tubulovillous adenoma of the gastroesphageal junction (FAP) that was later diagnosed as gastric cancer. Three of these four patients were diagnosed gastric cancer with further workup (2 FAP, 1 AFAP). The gastric phenotype was not significantly different between FAP and AFAP patients.

### Factors associated with of duodenal and gastric polyposis when considering all cases together regardless of *APC* mutation location

Gender was not associated with duodenal or gastric polyposis (Table 3). Age at endoscopy was modestly associated with gastric polyposis (*p* = 0.02) suggesting that gastric polyp severity is associated with an increase in age (Table 3). There is a mild association of advancing age on duodenal polyp severity as well, but this is also not statistically significant (*p* = 0.09).

**Table 3:** Gender and age at time of endoscopy as predictors of duodenal or gastric polyps

	Sum diameter of duodenal polyps			Gastric polyp count	
All participants (*n* = 150)					
Gender: Male % (n)	42% (*n* = 62)	Chi sq. = 0.63	*p*-value = 0.4259	Chi sq. = 0.28	*p*-value = 0.5991
Age at Endoscopy	Median (Q1-Q3)	Spearman = 0.14	*p*-value = 0.0873	Spearman = 0.19	*p*-value = 0.0191
Exclude current smokers (*n* = 128)					
Gender: Male % (n)	41% (*n* = 53)	Chi sq. = 0.3685	*p*-value = 0.5438	Chi sq. = 0.3022	*p*-value = 0.5825
Age at Endoscopy	Median (Q1-Q3) Spearman = 0.12	*p*-value = 0.1643	Spearman = 0.21	*p*-value = 0.0197	
Exclude NSAID use in past 3 months (*n* = 133)					
Gender: Male % (n)	41% (*n* = 55)	Chi sq. = 1.0689	*p*-value = 0.3012	Chi sq. = 0.3216	*p*-value = 0.5706
Age at Endoscopy	Median (Q1-Q3) Spearman = 0.12	*p*-value = 0.1643	Spearman = 0.21	*p*-value = 0.0197	
Exclude non-genetic testers (*n* = 140)					
Gender: Male % (n)	41% (*n* = 55)	Chi sq. = 1.0689	*p*-value = 0.3012	Chi sq. = 0.3216	*p*-value = 0.5706
Age at Endoscopy	Median (Q1-Q3) Spearman = 0.12	*p*-value = 0.1643	Spearman = 0.21	*p*-value = 0.0197	

The statistics for gender and advancing age are not affected when the current smokers, NSAID use in the past 3 months or lack of genetic diagnosis are excluded (Table 3).

We were interested in evaluating if the severity of colonic phenotype was a predictor of the severity of duodenal polyp burden. Because most patients had undergone colectomy at different ages and stages of colonic disease, an accurate measure was not possible. We did, however, use the age at time of colectomy as a rough proxy for colonic severity and examined this for corresponding duodenal severity (Fig. 1). The cohort consisted of 110 subjects with a colectomy (25 juvenile at age 8–17 years; 38 young adult at age 18–30 years; 29 adult at age 31–40 years and 18 mature adult at age 41 or older) and 40 without. As shown in Fig. 1, no clustering of duodenal polyp burden is observed with the 5 subgroupings of colectomy age. Patients who undergo colectomy at similar ages do not have similar duodenal polyp burden.

**Fig. 2:**
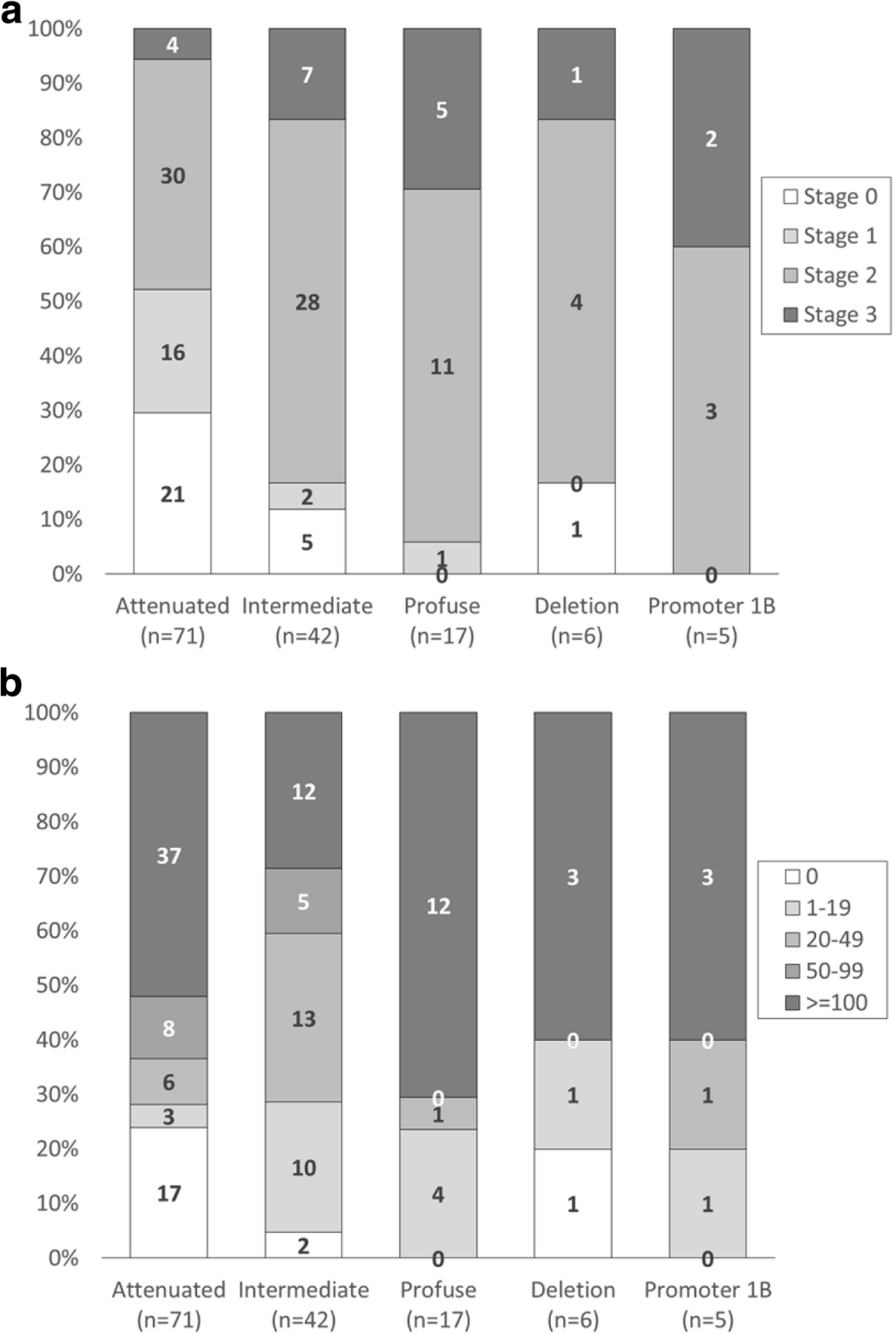
Modified Spigelman stage and gastric polyp number relative to *APC* mutation location Patients were divided into 5 groups based on the location of the *APC* mutation: Attenuated polyposis (*n* = 71), patients with mutations consistent with attenuated FAP (5′ to c.532, exon 9 alternative splice site c.936-c.1236, intron 9 and 3′ to c. 4785). Intermediate polyposis (*n* = 42); Profuse polyposis (*n* = 17) patients with mutations consistent with profuse colonic polyposis (c.3750-c.4392); Deletion of multiple APC exons (*n* = 6) and deletion of promoter 1B (*n* = 5). Figure shows percent and number of patients in each APC mutation group by (Panel **a**) modified Spigelman stage of duodenal polyposis or (Panel **b**) gastric polyp number. Gastric polyps were estimated as described in methods and set at a maximum of 100

The *APC* mutation location has been correlated with profuse, intermediate and attenuated colonic polyposis but the association between germline *APC* genotype and the severity of upper gastrointestinal polyposis is controversial (reviewed by [14]). Thus, we also examined the duodenal and gastric polyp phenotype based on these criteria. Patients were divided into 5 groups based on the location of the mutation: profuse (c.3750-c.4392), attenuated (5′ to c.531, exon 9 alternative splice site c.936-c.1236 and 3′ to c. 4785), intermediate (the remainder of coding mutations), large multiple exon deletions and promoter 1B deletions (Fig. 2) [18]. Although the attenuated FAP patients have overall lower duodenal polyp staging, examples of zero to moderate duodenal polyposis are observed (Fig. 2a). This is also generally true of the other 4 classes of mutations. The number of gastric polyps also varies within all 5 classes of mutations (Fig. 2b). It is notable, though, that none of the individuals with the “profuse” or “promoter 1B” mutations are free of duodenal or gastric polyps. The varied upper GI phenotype, even between patients with the same mutation, suggests that other genetic, epigenetic, or environmental factors modify the upper gastrointestinal phenotype.

## Discussion

The colonic phenotype and clinical management can be quite different depending on the *APC* mutation location [1]. Previous reports describe that upper gastrointestinal polyps are common in both classical FAP and AFAP, but, up to this point, no large prospective studies have addressed the similarities and differences in the upper GI phenotype of individuals diagnosed with FAP [14]. Here we show that the duodenal phenotype is milder in patients with an *APC* mutation consistent with AFAP, but there is no difference in the gastric phenotype. These general findings are similar to a previous studies from Japan [11, 19]. Although we did not specifically examine *Helicobacter pylori* infection as risk factor for the gastric phenotype, it is an established risk factor for gastric adenomas and cancer. Interestingly, *Helicobacter pylori* infection is found to be inversely correlated with fundic gland polyps in FAP as well as non-FAP patients [20, 21]. Because of the elevated risk of cancer, our standard practice is to test and treat in patients with FAP whenever *Helicobacter pylori* infection is suspected.

Although the duodenal polyp burden is milder in AFAP patients, a wide range of phenotype is observed. In this study, 48% of AFAP patients were found to have modified Spigelman stage II or higher duodenal polyposis. There are also examples of zero to moderate duodenal polyp burden in individuals harboring the identical mutation predisposing to AFAP (*APC* c.426_427delAT) as well as FAP (*APC* c.3225 T > A). The age at which an individual undergoes colectomy, which can be considered an indicator of colonic phenotype severity, does not predict duodenal polyp severity suggesting that factors that drive the onset of severity of colonic polyposis are different than factors that underlie severity of duodenal polyposis.

Because this study was associated with a chemoprevention trial, the biases and limitations must be considered. First, prescreening of subjects for eligibility introduced biases, in particular exclusion of those with a severe upper GI polyposis resulting in previous surgery. Fourteen percent of individuals excluded had severe duodenal polyposis or had previously undergone a duodenectomy; 85% of these patients were classified as FAP. Thus inclusion of these individuals would result in a much greater proportion of FAP patients having stage IV duodenal polyposis. Indeed, only 1% of our cohort had Spigelman stage IV duodenal polyposis in contrast to 7% of the cohort that is the basis of clinical recommendations [4]. The inverse, exclusion of those with no evidence of duodenal polyposis, was also true. If individuals had zero duodenal polyps documented in recent endoscopies, they were generally not pursued for enrollment. This, however, was only 3% of the excluded individuals and equally represented between FAP and AFAP. Even so, we find that 7% of our FAP patients and 30% of our AFAP patients had zero duodenal polyps in contrast to 34% of the FAP patients described by Bulow et al. [4]. Multiple factors may account for this difference between studies, including use of multiple passes plus side viewing endoscopy (this study) versus forward viewing endoscopy (Bulow), previous removal of duodenal polyps, and proportion of FAP versus AFAP patients (not documented in Bulow). Another limitation was use of modified Spigelman criteria. Because polyps were not removed for histologic analysis unless they were of clinical concern, we modified the Spigelman criteria by making assumptions as to the histology of polyps based on published size/histology correlations [16, 17]. Two additional variables that may have biased the results were specifically examined. This included individuals who had been on NSAIDs during the past 3 months and current smokers. Exclusion of individuals with these two variables did not change the results. Although we had limitations involving the distribution of severity of polyps and making assumptions as to the histology, we are able to show that the duodenal phenotype is different based on genotype, but gastric phenotype is not, in FAP patients.

## Conclusion

We provide here evidence to support current guidelines for upper GI surveillance which recommend starting at age 20 to 25 with a frequency ranging from 3 to 48 months as determined by the extent of duodenal polyposis (NCCN Guidelines Version 2.2015). This guideline is based solely on individual findings. Duodenal polyp burden is milder on average in individuals with *APC* mutations associated with AFAP versus FAP, but severe duodenal polyposis is observed in AFAP. Standard surveillance should occur and should not be modified based on the specific mutation, the colonic phenotype, or even the presentation of other affected family members.

## Abbreviations

AFAP: attenuated familial adenomatous polyposis
APC: Adenomatous polyposis coli
CHRPE: congenital hypertrophy of the retinal pigment epithelia
EGDs: esophagogastroduodenoscopies
FAP: familial adenomatous polyposis
GAPPS: gastric adenocarcinoma and proximal polyposis of the stomach
GI: gastrointestinal
NSAID: nonsteroidal anti-inflammatory drug
